# Association of inflammation geriatric nutritional risk index with all-cause and CVD mortality in patients with osteoarthritis

**DOI:** 10.3389/fnut.2025.1616413

**Published:** 2025-08-06

**Authors:** Gaohua Cao, Yunxuan Zou, Lei Tan, Yuanyuan Zhou, Shaodong Xie, Ke Jie, He Chen

**Affiliations:** ^1^The Eighth Clinical Medical College of Guangzhou University of Traditional Chinese Medicine, Foshan, China; ^2^Foshan Traditional Chinese Medicine Hospital, Foshan, China

**Keywords:** osteoarthritis, geriatric nutritional risk index, all-cause mortality, cardiovascular mortality, NHANES, nutritional status

## Abstract

**Background:**

Osteoarthritis (OA) is a prevalent chronic disease affecting the elderly, with significant implications for morbidity and mortality. The geriatric nutritional risk index (GNRI) score is a tool for assessing the nutritional status of elderly patients and has been linked to mortality outcomes in various chronic diseases. However, its relationship with mortality in OA patients remains underexplored.

**Objective:**

To evaluate the association between GNRI levels and all-cause and cardiovascular mortality in patients with OA, using data from NHANES (1999–2018).

**Methods:**

This cohort study included 3,023 OA patients. GNRI was calculated using serum albumin levels and body weight data. Mortality outcomes were tracked until December 31, 2019, linking NHANES data with the National Death Index. Statistical analyses included Cox proportional hazards models, Kaplan–Meier survival curves, and subgroup analyses.

**Results:**

High GNRI was significantly associated with reduced all-cause and cardiovascular mortality, even after adjusting for potential confounders. The relationship between GNRI and all-cause mortality was nonlinear, with the most significant protective effect observed at GNRI values below 130.55. Subgroup analysis revealed that the effect of GNRI on mortality risk was more pronounced in individuals aged 65 or older and non-Hispanic White people.

**Conclusion:**

GNRI is a valuable prognostic tool for predicting mortality risk in OA patients. This study highlights the importance of incorporating nutritional assessments into the management of OA, particularly for high-risk populations. Future research should explore the potential benefits of nutritional interventions based on GNRI levels in reducing mortality among OA patients.

## 1 Introduction

Osteoarthritis (OA) is a common, chronic degenerative joint disorder characterized by progressive cartilage deterioration and inflammation of periarticular structures, manifesting clinically as joint pain, stiffness, and reduced mobility (1–3). Globally, OA affects an estimated 8% of the population and is a major cause of disability, particularly in older adults (4, 5). With the growing aging population, the burden of OA is increasing, contributing not only to morbidity but also to elevated mortality risks (6). Recent studies have identified multiple risk factors influencing OA progression, including metabolic, inflammatory, mechanical, and nutritional components (7–10). Among these, nutritional status has emerged as a potentially modifiable factor, yet remains underexplored in OA management. Malnutrition, particularly protein-energy deficiency, is prevalent among older adults with chronic conditions and has been linked to frailty, sarcopenia, systemic inflammation, and impaired immune response—all of which may exacerbate OA symptoms and outcomes. For instance, Win et al. (11) reported a high prevalence of malnutrition among elderly individuals receiving home-based care for chronic diseases, including OA, further supporting the need for systematic nutritional assessment in this population. Zhao et al. (12) demonstrated that higher dietary inflammatory index scores were associated with frailty in OA patients, and Li et al. (13) found that lower serum albumin levels correlated with increased mortality in OA populations. Despite these findings, comprehensive nutritional screening tools are rarely integrated into routine OA care.

Among the various nutritional assessment tools available—such as the Mini Nutritional Assessment (MNA), Subjective Global Assessment (SGA), and Nutrition Risk Screening 2002 (NRS-2002)—the geriatric nutritional risk index (GNRI) score has emerged as a simple, objective, and reproducible index that does not rely on subjective reporting or dietary recall (14). The GNRI is a validated measure incorporating serum albumin and body weight relative to ideal weight, offering a composite assessment of nutritional risk in older adults. GNRI has been shown to predict mortality in various chronic diseases, including heart failure, chronic kidney disease, and stroke (15–17). However, its application in patients with osteoarthritis (OA) remains limited, and its prognostic value in relation to all-cause and cardiovascular mortality has not been thoroughly investigated in this population. In recent years, the relationship between nutritional status and adverse outcomes in patients with OA—particularly knee OA—has received growing attention. Several studies have suggested that poor nutritional status may contribute to increased mortality risk among OA patients (18). In addition, low GNRI scores have shown clinical significance in other musculoskeletal conditions; for example, in young male patients with rheumatoid arthritis, GNRI was found to be an independent risk factor for abnormal bone metabolism (19). These findings suggest a potential role for GNRI in identifying vulnerable subgroups within the OA population. To address this knowledge gap, the present study aims to evaluate the association between GNRI and mortality outcomes in patients with OA using data from the National Health and Nutrition Examination Survey (NHANES) (20). We hypothesize that lower GNRI scores are independently associated with increased risk of all-cause and cardiovascular death, and that this relationship may differ by demographic and clinical subgroups. This study aims to provide evidence supporting the integration of nutritional assessment into OA management and risk stratification (21). Although NHANES provides a robust and nationally representative dataset, the diagnosis of OA in this study is based on self-reported physician confirmation, which may introduce potential recall bias or misclassification. This diagnostic approach, while widely used in epidemiologic research utilizing NHANES, lacks radiographic or clinical validation. Nevertheless, previous studies have reported reasonable agreement between self-reported OA and clinical diagnosis in older adults, supporting its use in large-scale population analyses. This limitation is acknowledged and further addressed in the Methods and Discussion sections.

Meanwhile, the relationship between inflammatory status and GNRI levels has received increasing attention and may play an important role in the risk of all-cause and cardiovascular disease (CVD) mortality in OA patients. For example, a higher systemic immune inflammation index has been shown to be significantly associated with all-cause mortality in OA patients, suggesting a key role of inflammation in OA disease progression (18, 22). It has also been found that elevated levels of GNRI may be associated with lower all-cause and CVD mortality (6), further supporting the potential benefits of improved nutritional status on the long-term prognosis of OA patients.

Although studies have shown an association between dietary factors and OA in the elderly (21), there is still a lack of systematic investigation of the relationship between GNRI and mortality risk in patients with OA. This study aimed to further evaluate the potential association between GNRI levels and all-cause and cardiovascular mortality in patients with OA to provide new perspectives for risk assessment and intervention in this population.

## 2 Methods

### 2.1 Study design and population

**Figure 1:**
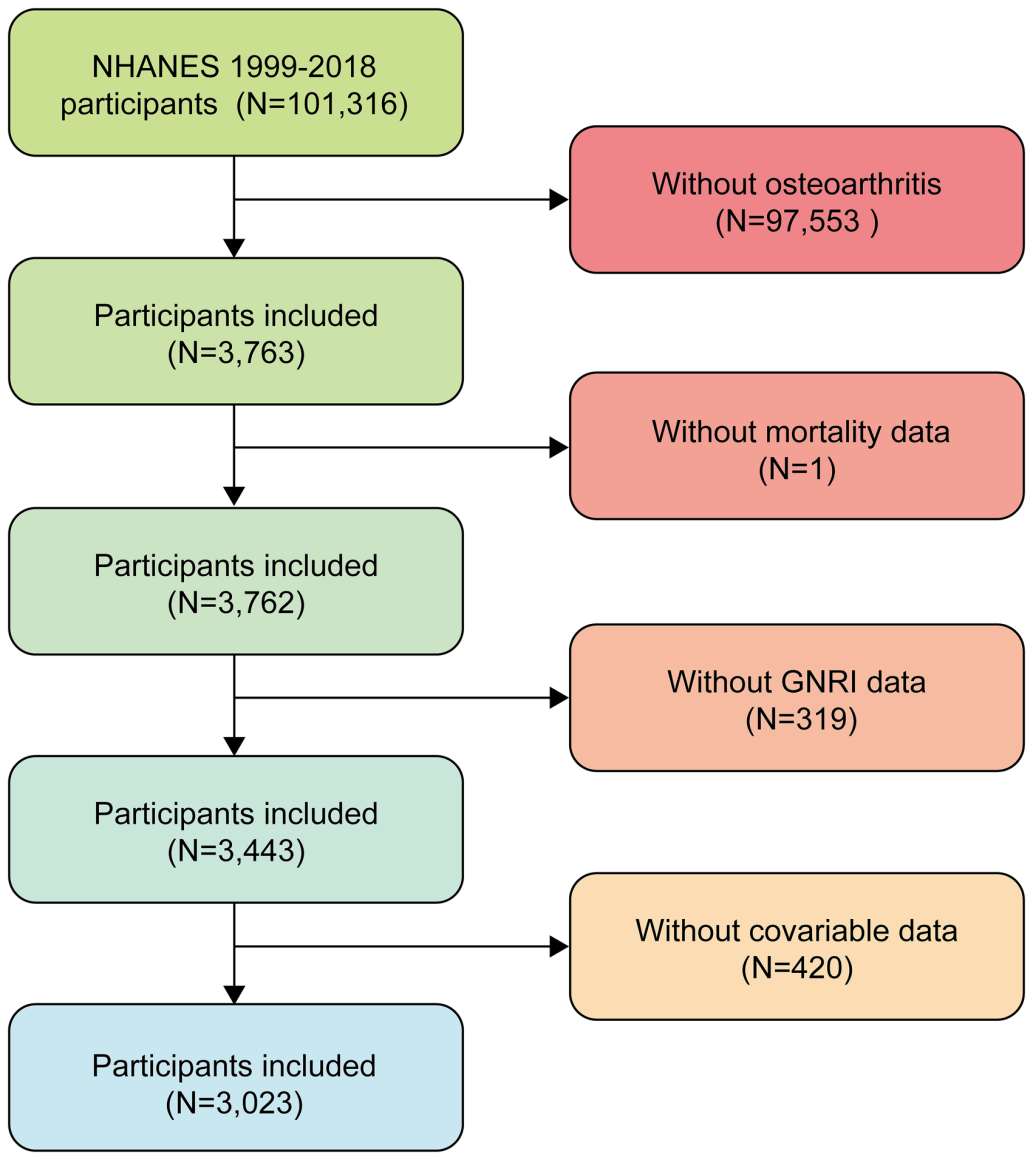
Flowchart of selected samples.

This study used data from NHANES 1999–2018, which included a total of 101,316 participants. First, we excluded participants who denied having osteoarthritis (*N* = 97,553), leaving 3,763 participants. Then, 319 participants with missing GNRI data, 420 participants with missing covariate data, and 1 participant with missing mortality data were further excluded, resulting in the inclusion of 3,023 eligible participants. The participant screening process for this study is shown in Figure 1. The survey uses a multistage stratified probability sampling design to systematically collect data on the health and nutritional status of noninstitutionalized U.S. residents. This study used appropriate weights for weighting.

Study participants were selected according to the following criteria: (1) self-reported physician-diagnosed osteoarthritis; (2) available mortality follow-up information; and (3) complete data for calculating the GNRI. Individuals with missing key covariate data were excluded. All participants provided informed consent. To evaluate OA, participants were asked the following two questions during the NHANES interview: (1) “Has a doctor or other health professional ever told you that you have arthritis?” and (2) “What type of arthritis was it?” Participants who responded “osteoarthritis or degenerative arthritis” were classified as having OA. Although this self-report approach does not include radiographic or clinical validation, it has been used in prior population-based studies and is consistent with NHANES methodology. Nevertheless, we recognize the inherent limitations of this classification method and address them in the discussion.

### 2.2 Definition of GNRI

In this study, the formula was used to calculate the GNRI: GNRI = 1.489 × serum albumin (g/L) + 41.7 × (measured weight/ideal weight). The measured weight covered self-reported, actual or medical record data, and the ideal weight was derived based on the standard Body mass index (BMI) (22 kg/m^2^) with the formula: height (m)^2^ × 22. Based on the calculation results, the subjects were divided into low GNRI and high GNRI groups for comparative analysis.

### 2.3 Outcomes

The primary outcomes were all-cause mortality and CVD mortality. Mortality information was obtained by linking NHANES data with the National Death Index (NDI) through December 31, 2019. Cardiovascular disease deaths were determined according to International Classification of Diseases, 10th Revision (ICD-10) codes I00–I09, I11, I13, I20–I51, and I60–I69. All participants were followed from the baseline survey date until the occurrence of death or the end of follow-up. To facilitate subgroup analysis, we further categorized GNRI scores into “low” and “high” groups based on the data-derived inflection point (130.55) identified through two-piecewise Cox regression modeling.

### 2.4 Covariables

To reduce the influence of potential confounding factors, we collected the following covariates: demographic characteristics including age, sex, race/ethnicity (Non-Hispanic Black, Mexican American, other Hispanic, Non-Hispanic White, and other race), education level (<high school, high school or equivalent, >high school), marital status (married/living with partner, widowed/divorced/separated, never married), and family income-to-poverty ratio; health behaviors including smoking status (never smoker, former smoker, current smoker) and alcohol consumption status (non-drinker, drinker); and medical history and clinical characteristics including hypertension (systolic blood pressure ≥140 mmHg or diastolic blood pressure ≥90 mmHg or self-reported or use of antihypertensive medications), diabetes (based on physician diagnosis, fasting blood glucose ≥126 mg/dL, hemoglobin A1c ≥6.5%, or use of glucose-lowering medications), and chronic kidney disease (estimated glomerular filtration rate <60 mL/min/1.73m^2^ or ACR >30 mg/g). Laboratory parameters included total cholesterol and BMI.

### 2.5 Statistical analysis

In this study, the data were analyzed using a stratified statistical strategy: baseline characteristics were presented by survival status, continuous variables were described as mean ± standard error, and categorical variables were described as component ratios. Comparisons between groups were made using ANOVA/non-parametric tests (continuous variables) versus chi-square/exact tests (categorical variables), respectively. Differences in survival between groups were assessed by Kaplan–Meier survival curves with time-series tests, and Cox proportional risk models were applied to calculate all-cause and cardiovascular mortality risk ratios (HR) and their 95% confidence intervals (CI).

Kaplan–Meier survival curves and log-rank tests assessed survival probability differences across GNRI groups. Cox proportional hazards regression models estimated the association between GNRI and all-cause and cardiovascular mortality, expressed as hazard ratios (HRs) with 95% confidence intervals (CIs). Adjustments were made in three models: Model 1 was unadjusted; Model 2 included adjustments for age, sex, race/ethnicity, education, marital status, and BMI; Model 3 further adjusted for hypertension, diabetes, chronic kidney disease, cancer, and total cholesterol levels.

To explore the non-linear relationship between GNRI and mortality risk, smooth curve fitting and threshold effect analyses identified potential inflection points. Notably, the identified inflection point of GNRI = 130.55 in our study is consistent with thresholds reported in prior research involving older adults and chronic disease populations. For example, Yenibertiz et al. (23) originally categorized GNRI <92 as major risk, 92–98 as moderate risk, 98–100 as low risk, and >100 as no risk, though later studies have found that a threshold around 130 better stratifies mortality risk in non-hospitalized and community-based cohorts. Our finding reinforces the emerging view that GNRI scores below 130 may indicate a nutritionally vulnerable state associated with increased mortality risk, particularly in elderly OA patients. In addition, subgroup analyses evaluated the consistency of this relationship across age, sex, BMI, lifestyle factors, and comorbidities. All analyses were performed using R (version 4.2.0) and EmpowerStats (version 4.2), with *p*-values <0.05 considered statistically significant.

## 3 Results

### 3.1 Characteristics of the study population

Table 1 demonstrates the baseline characteristics of patients with OA, with group comparisons based on survival status. After weighting, the overall representative sample included 14,065,757 individuals, of which 11,127,967 were in the survival group and 2,937,790 were in the death group. The survival group had a significantly higher GNRI (155.52 ± 0.45) compared to the death group (149.83 ± 0.79; *p* < 0.0001). Patients in the death group were significantly older (71.22 ± 0.55 vs. 58.39 ± 0.38; *p* < 0.0001), had lower BMI (27.61 ± 0.24 vs. 29.04 ± 0.14; *p* < 0.0001), and had a lower income-to-poverty ratio (2.67 ± 0.08 vs. 3.55 ± 0.05; *p* < 0.0001). Total cholesterol was also slightly lower in the death group (196.19 ± 1.64 vs. 201.15 ± 1.49; *p* = 0.0265). In terms of gender, the death group had a higher proportion of males (52.59% vs. 39.29%; *p* < 0.0001). Marital status also differed significantly, with more married or cohabiting individuals in the survival group (70.19% vs. 58.46%; *p* < 0.0001), while the death group had a higher proportion of widowed, divorced, or separated participants (35.28% vs. 22.06%). Educational attainment was significantly different, with the survival group showing a higher percentage of individuals with education above high school (70.46% vs. 49.61%; *p* < 0.0001). Regarding alcohol consumption, drinkers were more prevalent in the survival group (80.20% vs. 69.31%; *p* < 0.0001), and smoking behavior also varied: former smokers were more common in the death group (49.07% vs. 35.67%; *p* < 0.0001), while never-smokers were more common in the survival group (48.10% vs. 35.67%; *p* < 0.0001). As for comorbidities, diabetes (24.74% vs. 18.42%; *p* = 0.0016), hypertension (62.07% vs. 44.63%; *p* < 0.0001), and chronic kidney disease (CKD) (28.52% vs. 11.30%; *p* < 0.0001) were all more prevalent in the death group compared to the survival group.

**Table 1:** Baseline characteristics of osteoarthritis patients according to survive status.

Characteristics	Overall (*n* = 14,065,757)	Survive (*n* = 11,127,967)	Death (*n* = 112,937,790)	*p*-value
GNRI	154.33 ± 0.40	155.52 ± 0.45	149.83 ± 0.79	<0.0001
Follow-up time (months)	103.56 ± 1.81	107.45 ± 2.22	88.81 ± 2.00	<0.0001
Age (years)	61.07 ± 0.35	58.39 ± 0.38	71.22 ± 0.55	<0.0001
BMI (kg/m^2^)	28.74 ± 0.12	29.04 ± 0.14	27.61 ± 0.24	<0.0001
Income to poverty ratio	3.36 ± 0.05	3.55 ± 0.05	2.67 ± 0.08	<0.0001
Total cholesterol (mg/dL)	200.11 ± 1.22	201.15 ± 1.49	196.19 ± 1.64	0.0265
Gender, %				<0.0001
Male	42.06%	39.29%	52.59%	
Female	57.94%	60.71%	47.41%	
Race/Ethnicity, %				0.091
Mexican American	2.01%	2.24%	1.13%	
Other Hispanic	2.24%	2.28%	2.08%	
Non-Hispanic White	88.14%	87.70%	89.81%	
Non-Hispanic Black	4.37%	4.13%	5.27%	
Other races	3.25%	3.65%	1.72%	
Marital status, %				<0.0001
Married/Living with partner	67.74%	70.19%	58.46%	
Widowed/Divorced/Separated	24.82%	22.06%	35.28%	
Never married	7.44%	7.75%	6.25%	
Education level, %				<0.0001
Less than high school	0.04	2.52%	9.34%	
High school or GED	29.95%	27.02%	41.05%	
Above high school	66.11%	70.46%	49.61%	
Drinking status, %				<0.0001
Yes	77.93%	80.20%	69.31%	
No	22.07%	19.80%	30.69%	
Smoking status, %				<0.0001
Current	16.03%	16.23%	15.26%	
Former	38.47%	35.67%	49.07%	
Never	45.51%	48.10%	35.67%	
Diabetes, %				0.0016
Yes	19.74%	18.42%	24.74%	
No	45.00%	46.44%	39.52%	
Borderline	35.26%	35.14%	35.74%	
Hypertension, %				<0.0001
Yes	51.73%	44.63%	62.07%	
No	48.27%	55.37%	37.93%	
CKD, %				<0.0001
Yes	14.90%	11.30%	28.52%	
No	85.10%	88.70%	71.48%	

### 3.2 Logistic regression analyses

Table 2 demonstrates the association between GNRI and mortality in two groups of OA patients, including all-cause mortality and CVD mortality. The results show that there is a statistically significant association between GNRI and mortality in OA patients, even after adjusting for multiple confounders. In terms of all-cause mortality, continuous GNRI was significantly negatively associated with mortality (Model 3: HR = 0.96, 95% CI: 0.95, 0.98, *p* < 0.0001). High GNRI was associated with a 22% reduction in all-cause mortality in patients with OA it is compared with the low GNRI group (Model 3: HR = 0.78, 95% CI: 0.61, 0.98, *p* = 0.0323). In terms of cardiovascular disease mortality, continuous GNRI was also significantly and negatively associated with mortality (Model 3: HR = 0.95, 95% CI: 0.93, 0.98, *p* < 0.0001). High GNRI was associated with a 32% reduction in cardiovascular mortality in patients with OA in the unadjusted model compared with the low GNRI group (Model 3: HR = 0.68, 95% CI: 0.51, 0.90, *p* = 0.0079). However, this negative correlation failed to remain significant in both the partial and fully adjusted models.

**Table 2:** Association between GNRI and mortality.

Outcomes	Model 1 [HR (95% CI)] *p*-value	Model 2 [HR (95% CI)] *p*-value	Model 3 [HR (95% CI)] *p*-value
All-cause mortality			
Continuous GNRI	0.99 (0.98, 0.99) < 0.0001	0.96 (0.95, 0.98) < 0.0001	0.96 (0.95, 0.98) < 0.0001
Low GNRI	Ref	Ref	Ref
High GNRI	0.70 (0.60, 0.81) < 0.0001	0.77 (0.62, 0.96) 0.0196	0.78 (0.61, 0.98) 0.0323
CVD mortality			
Continuous GNRI	0.99 (0.98, 1.00) 0.0045	0.95 (0.93, 0.98) 0.0003	0.95 (0.93, 0.98) 0.0001
Low GNRI	Ref	Ref	Ref
High GNRI	0.68 (0.51, 0.90) 0.0079	0.67 (0.43, 1.05) 0.0772	0.62 (0.38, 1.00) 0.0519

**Figure 2:**
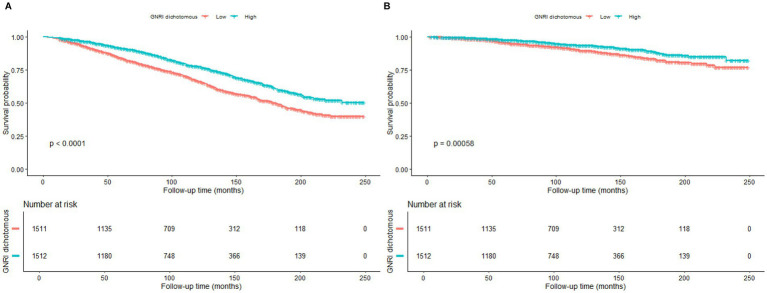
Kaplan–Meier survival curves for the relationship between GNRI and mortality. **(A)** All-cause mortality. **(B)** Cardiovascular disease mortality.

Figures 2A,B demonstrate the survival curves for GNRI in terms of all-cause mortality and CVD mortality in patients with OA. Figure 2A shows that there was a significant difference in survival probability between the high GNRI group and the low GNRI group in terms of all-cause mortality. The survival probability of the high GNRI group was higher than that of the low GNRI group as the follow-up time increased (*p* < 0.0001). Figure 2B similarly demonstrates the survival curve for CVD mortality, with a higher survival probability in the high-GNRI group than in the low-GNRI group (*p* = 0.00058). Over time, the high GNRI group consistently maintained a higher survival probability, further supporting the effect of GNRI on mortality in OA patients.

#### 3.2.1 Empirical analysis of nonlinear and threshold effects

**Figure 3:**
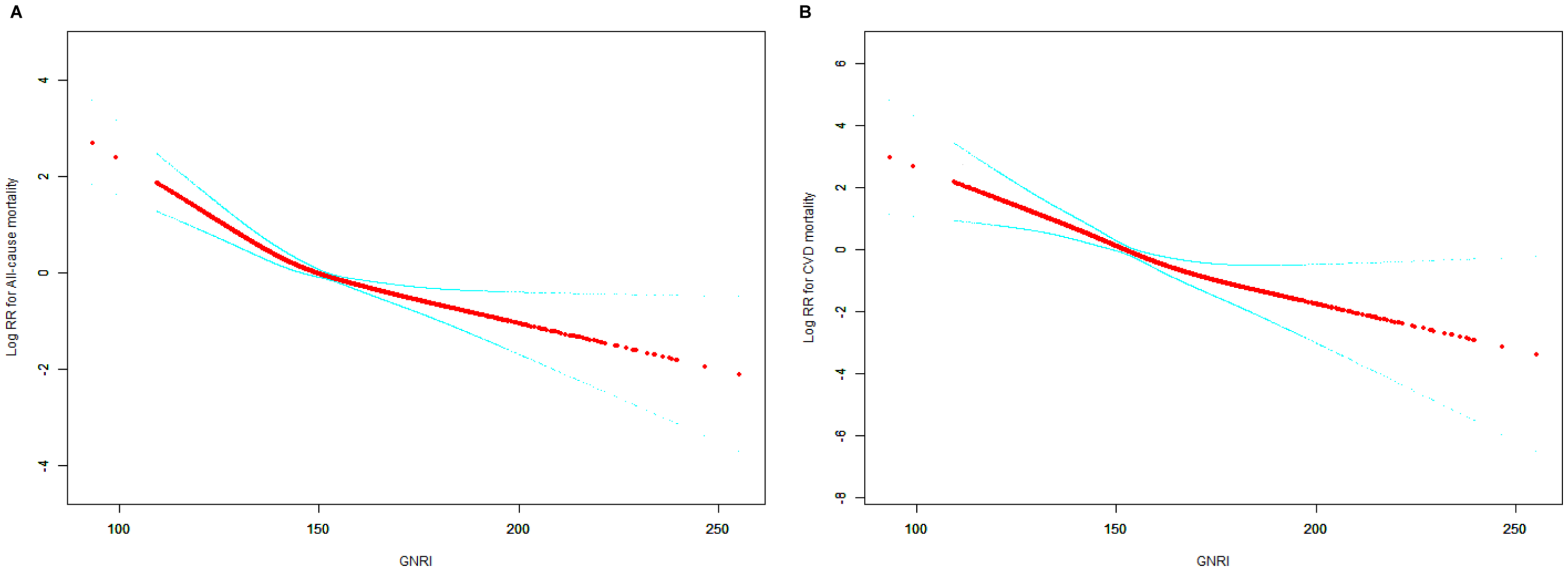
Smooth curve fitting for the relationship between GNRI and mortality. **(A)** All-cause mortality. **(B)** Cardiovascular disease mortality.

Figures 3A,B demonstrate the inverse relationship between GNRI and all-cause mortality and CVD mortality, i.e., the risk ratio (RR) increased significantly at lower GNRIs and leveled off at higher GNRIs.

Table 3 demonstrates two-stage linear modeling analysis revealed a turning point of 130.55 for all-cause mortality and (*p* < 0.001). Below the turning point, the association between GNRI and all-cause mortality was significant and remained significant beyond the turning point. However GNRI was linearly associated with CVD mortality (*p* for log-likelihood ratio = 0.086).

**Table 3:** Threshold effect analysis of GNRI score on mortality using two-piecewise Cox regression.

Description	All-cause mortality	CVD mortality
Fitting by the standard linear model	0.97 (0.96, 0.98) < 0.0001	0.95 (0.93, 0.98) 0.0001
Fitting by the two-piecewise linear model		
Inflection point	130.55	132.72
<Inflection point	0.92 (0.90, 0.94) < 0.0001	0.92 (0.88, 0.96) 0.0003
>Inflection point	0.97 (0.96, 0.99) < 0.0001	0.96 (0.93, 0.98) 0.0006
*p* for log-likelihood ratio	<0.001	0.086

### 3.3 Subgroup analysis

Table 4 demonstrates the subgroup analysis of the association between GNRI and all-cause mortality and CVD mortality in OA patients with different demographic and health-related factors. The analysis showed that the protective association between GNRI and all-cause mortality in OA patients was consistent across all subgroup classifications (*p* for interaction >0.05). The protective association between GNRI and CVD mortality in OA patients was also consistent across most subgroup classifications (*p* for interaction >0.05). However, age and race could significantly modify this association (*p* for interaction <0.05). Among those aged 65 years and older, higher GNRI was associated with a lower risk of CVD mortality (HR = 0.96, 95% CI: 0.94, 0.99, *p* = 0.0047); in subgroup analyses of race, the protective effect of the association between GNRI and CVD mortality varied across racial groups, and for non-Hispanic White people the GNRI-CVD mortality was significantly and negatively associated (HR = 0.96, 95% CI: 0.94–0.99, *p* = 0.0047), whereas for and Mexican American, non-Hispanic Black, other Hispanic, and other racial groups with OA, the protective effect of GNRI with CVD mortality risk was not significant; in terms of health, a higher GNRI in diabetes-prevalent groups was associated with a lower risk of CVD death was significantly associated (HR = 0.94, 95% CI: 0.92, 0.97, *p* = 0.0424).

**Table 4:** Subgroup analyses for the relationship between GNRI and mortality.

Outcomes	All-cause mortality		CVD mortality	
	HR (95% CI)	*p* for interaction	HR (95% CI)	*p* for interaction
Sex		0.3856		0.8419
Male	0.97 (0.96, 0.98)		0.95 (0.93, 0.98)	
Female	0.97 (0.95, 0.98)		0.95 (0.93, 0.98)	
Age		0.1708		0.0047
<65	0.96 (0.95, 0.98)		0.96 (0.94, 0.99)	
≥65	0.96 (0.95, 0.97)		0.94 (0.91, 0.96)	
BMI		0.0517		0.3085
<25	0.97 (0.96, 0.98)		0.96 (0.94, 0.99)	
≥25, <30	0.97 (0.96, 0.99)		0.98 (0.95, 1.00)	
≥30	0.99 (0.98, 1.00)		0.99 (0.97, 1.01)	
Race		0.7459		0.0279
Mexican American	0.97 (0.95, 1.00)		0.98 (0.93, 1.03)	
Other Hispanic	0.96 (0.93, 0.99)		0.90 (0.80, 1.00)	
Non-Hispanic White	0.97 (0.96, 0.98)		0.95 (0.93, 0.97)	
Non-Hispanic Black	0.97 (0.95, 0.98)		0.98 (0.95, 1.01)	
Other races	0.96 (0.93, 0.99)		1.03 (0.91, 1.15)	
Smoking behavior		0.1122		0.0701
Current	0.96 (0.94, 0.97)		0.94 (0.90, 0.98)	
Former	0.97 (0.96, 0.98)		0.96 (0.94, 0.98)	
Never	0.97 (0.95, 0.98)		0.94 (0.92, 0.97)	
Drinking behavior		0.2094		0.3543
Yes	0.97 (0.96, 0.98)		0.96 (0.93, 0.98)	
No	0.96 (0.95, 0.98)		0.95 (0.92, 0.97)	
Diabetes		0.9383		0.0524
Yes	0.97 (0.96, 0.98)		0.94 (0.92, 1.02)	
No	0.97 (0.96, 0.98)		0.97 (0.94, 0.99)	
Borderline	0.97 (0.95, 0.98)		0.95 (0.92, 0.98)	
Hypertension		0.3316		0.4451
Yes	0.97 (0.96, 0.98)		0.96 (0.93, 0.98)	
No	0.97 (0.95, 0.98)		0.95 (0.92, 0.97)	
CKD		0.3158		0.8489
Yes	0.97 (0.96, 0.98)		0.95 (0.93, 0.98)	
No	0.97 (0.95, 0.98)		0.95 (0.93, 0.98)	

## 4 Discussion

The present study revealed a significant negative correlation between GNRI and risk of death in patients with osteoarthritis. Higher GNRI levels were associated with reduced all-cause and cardiovascular mortality, and the relationship between GNRI and all-cause mortality was found to be nonlinear: the protective effect of GNRI was most pronounced at thresholds below 130.55. This negative association remained consistent across most subgroups. These findings emphasize the importance of incorporating nutritional assessment into the management of osteoarthritis, especially in high-risk populations with low GNRI values, to reduce the risk of mortality and improve clinical outcomes. GNRI has a predictive value for the prognosis of patients with osteoarthritis.

Available evidence suggests that GNRI has significant prognostic value in different clinical scenarios, a finding that is consistent with previous studies on the influence of nutritional status on survival outcomes in the elderly population (22, 24). Several clinical observations have validated the predictive efficacy of this index for mortality risk in elderly patients. It is worth noting that the GNRI threshold of 98 points can serve as an important threshold for risk stratification in specific disease groups: the risk of death in the group of patients undergoing transcatheter aortic valve replacement with GNRI <98 was 44% higher than in the normal group (25); the all-cause mortality rate of elderly stroke patients with abnormalities of this index was 3.64 times higher than that of the normal population (26). Guo and other scholars further found that in patients with osteoporosis, individuals with GNRI levels in the lowest tertile range had a 60–100% increased risk of death (27). In addition, this index is equally useful as an early warning for adverse clinical regression of common chronic diseases in the elderly such as chronic obstructive pulmonary disease, chronic kidney disease and cirrhosis of the liver (28–30). Osteoarthritis is a common chronic disease affecting the health of the elderly, and early intervention is essential to delay the condition. Although GNRI is widely used to assess the nutritional status and prognosis of older adults, its applicability in patients with osteoarthritis still needs to be explored. This study demonstrates that GNRI as a nutritional indicator can effectively identify patients at high risk of osteoarthritis, provide a basis for clinical intervention, and help optimize nutritional intervention and mortality risk management in patients by regularly monitoring GNRI.

The significant negative correlation between GNRI levels and all-cause and CVD mortality in patients with osteoarthritis over a mean follow-up of 8.5 years may reflect the profound impact of nutritional status on the health of OA patients. Low GNRI scores usually indicate malnutrition or protein-energy depletion (31), and malnutrition is a common health problem among OA patients (2, 32). Malnutrition not only leads to decreased immune function and muscle strength, but may also exacerbate the inflammatory response (33), all of which can increase the risk of infections, the probability of cardiovascular disease and other complications, and thus raise the risk of death (11, 34). In addition, patients with OA often face long-term chronic pain and dysfunction, which can lead to reduced activity levels and weight loss, which in turn worsens their nutritional status (35). Low GNRI may also be associated with an imbalance in the body fat-to-muscle ratio, which increases the risk of muscle weakness and debility, which is a significant predictor of increased mortality (36, 37). From a mechanistic perspective, poor nutritional status may accelerate OA-related mortality risk through several biological pathways. Malnutrition—particularly hypoalbuminemia and inadequate protein-energy intake—can impair immune competence, reduce antioxidant defenses, and promote systemic inflammation. This chronic inflammatory state, often referred to as “inflammaging,” plays a central role in both OA pathogenesis and cardiovascular disease progression. Elevated levels of inflammatory cytokines such as IL-6, TNF-α, and CRP have been associated with cartilage degradation, endothelial dysfunction, and metabolic dysregulation. In OA patients, undernutrition may further contribute to sarcopenia and muscle weakness, reducing physical activity levels and increasing vulnerability to falls, frailty, and hospitalization. These interrelated processes—nutritional depletion, inflammation, functional decline—may create a vicious cycle that heightens the risk of both all-cause and CVD mortality. Therefore, GNRI not only reflects nutritional reserves but may also serve as an indirect marker of inflammatory burden and physiological resilience in this population. Therefore, the association between GNRI, as a comprehensive indicator of nutrition, body weight and activity level, and the risk of death provides an important basis for clinical management of the nutritional status of patients with OA.

In this study, we found an L-shaped relationship between GNRI and all-cause mortality in patients with OA, which is consistent with the findings of Lim and Nam (38). The protective effect was most significant when the GNRI was below 130.55. This suggests that the effect of nutritional interventions is more significant at poorer levels of nutrition. When the GNRI is below these thresholds, patients have reduced immune function and disease resistance and increased susceptibility, leading to an increased risk of death. Low GNRI may also be associated with long-term chronic inflammation, which in turn exacerbates cardiovascular events and metabolic syndrome, creating a vicious cycle (39, 40). However, when GNRI exceeds these thresholds, the protective effects of further increases in GNRI plateau, suggesting that nutritional interventions have a “range of effectiveness” beyond which improvements diminish.

The results of the subgroup analyses showed that the association between GNRI and CVD mortality varied across age and ethnic groups. In particular, the association between GNRI and CVD mortality was more significant in younger patients <65 years of age. This may be related to the fact that younger patients have a lower general health status and chronic disease burden, and thus they are more responsive to nutritional interventions and lifestyle changes (41). In addition, the non-Hispanic Black group showed a stronger negative correlation between GNRI and CVD mortality, which may be related to socioeconomic status, nutritional intake, and health inequality factors in this group. Studies have shown that groups with lower socioeconomic status tend to be at higher risk for malnutrition (42), as well as more susceptible to chronic diseases and poor health behaviors (43), and thus the GNRI has a stronger prognostic value as a nutritional assessment tool in these groups. These results emphasize the importance of considering individual age and ethnic differences in clinical practice for accurate management of nutritional status and mortality risk in patients with OA.

These subgroup differences may reflect deeper biological and sociodemographic mechanisms. For instance, age-related immune decline and chronic low-grade inflammation (“inflammaging”) may amplify the prognostic impact of malnutrition in older adults, thereby strengthening the association between low GNRI and CVD mortality in this subgroup. Similarly, racial disparities may stem not only from genetic or metabolic differences, but also from unequal access to nutrition, healthcare resources, or preventive services. Non-Hispanic White individuals, who showed a significant inverse association, may have greater access to early-stage nutritional interventions or chronic disease management programs. In contrast, minority populations may face higher levels of food insecurity, healthcare underutilization, and chronic stress, which could attenuate or obscure the protective effects of higher GNRI. These findings underscore the need for culturally and demographically tailored nutrition assessments and interventions to reduce health disparities in OA populations.

The strength of this study lies in its relevance and clinical guidance. First, the study revealed the association between GNRI and mortality risk, emphasizing the importance of incorporating nutritional assessment in the clinical management of OA patients, especially for high-risk groups. Second, the study found a nonlinear relationship between GNRI and mortality risk and identified specific thresholds that provide guidance for clinical personalized health management. Finally, by analyzing subgroups of different age and ethnic groups, the study provides a basis for developing more precise interventions for specific high-risk groups.

However, there are some shortcomings in this study. First, as an observational cohort study, it is subject to residual confounding and cannot establish causality between GNRI and mortality outcomes. Second, OA diagnosis was based on self-reported physician diagnosis, which may be vulnerable to recall bias and potential misclassification, especially without radiographic confirmation. Third, NHANES does not collect detailed information on OA severity, joint involvement, or clinical symptoms, which may influence mortality risk and the nutritional status of participants. Fourth, data on nutritional interventions, dietary intake, or supplementation during follow-up were not available, limiting our ability to evaluate the effect of specific nutrition-related treatments. Finally, although we adjusted for multiple covariates, residual confounding may still be present.

## 5 Conclusion

This study demonstrated a significant negative association between the GNRI and the risk of all-cause mortality and cardiovascular disease CVD mortality in patients with OA. Higher levels of the GNRI were associated with a reduced risk of mortality, and the effects of nutritional interventions were more pronounced below a certain threshold. Subgroup analyses showed that the protective effect of GNRI on CVD death was particularly significant in certain populations, such as older adults and non-Hispanic White people. These findings emphasize the importance of incorporating nutritional assessment in clinical management, especially for high-risk patients. Regular monitoring and intervention based on GNRI levels can help to reduce mortality risk and improve clinical outcomes in patients with OA.

## Data Availability

Publicly available datasets were analyzed in this study. This data can be found here: the survey data are publicly available on the internet for data users and researchers throughout the world (www.cdc.gov/nchs/nhanes/).
